# Predictors of antenatal care service utilization among women of reproductive age in Somalia: a systematic review

**DOI:** 10.3389/frph.2026.1785888

**Published:** 2026-04-13

**Authors:** Ahmed Mohamed Dirie, Nur Rashid Ahmed, Shafie Abdirahman Dirie, Osman Abubakar Fiidow, Kassim Abdi Jimale, Abdirahman Mohamud Mohamed

**Affiliations:** 1 Faculty of Health Science, Salaam University, Mogadishu, Somalia; 2 Faculty of Medicine and Health Sciences, Jamhuriya University of Science and Technology, Mogadishu, Somalia

**Keywords:** antenatal care, predictors, Somalia, utilization, women of reproductive age

## Abstract

**Purpose:**

Antenatal care (ANC) is a cornerstone of maternal and neonatal health, yet its utilization remains suboptimal in Somalia, contributing to high maternal and perinatal morbidity and mortality. Understanding the predictors of ANC utilization is essential for designing effective interventions. This systematic review aimed to identify predictors of antenatal care service utilization among women of reproductive age in Somalia.

**Materials and methods:**

A systematic search of PubMed, Google Scholar, Medline, CINAHL, EMBASE, and African Journals Online was conducted for studies published between 2010 and 2025. Quantitative studies reporting predictors of ANC utilization using multivariable analysis were included. Study selection and reporting followed PRISMA guidelines. Methodological quality was assessed using standardized appraisal tools. The review protocol was registered in PROSPERO (registration number: CRD420251244002).

**Results:**

Nine studies met the inclusion criteria. ANC utilization in Somalia was influenced by a combination of sociodemographic, obstetric, knowledge-related, and health system factors. Consistently reported predictors included maternal education, place of residence, household wealth index, marital status, pregnancy intention, maternal age, and exposure to mass media. Early gestational age at first visit, gravida status, knowledge of ANC, distance to health facilities, accessibility of services, and perceived health worker attitudes were also significant predictors.

**Conclusion:**

Antenatal care utilization in Somalia is shaped by complex and interrelated individual, socioeconomic, and health system factors. Targeted interventions focusing on female education, community awareness, early ANC initiation, and improved access to quality maternal health services are critical to increasing ANC utilization and improving maternal and neonatal outcomes.

**Systematic Review Registration:**

https://www.york.ac.uk/inst/crd, PROSPERO registration number, CRD420251244002.

## Introduction

Antenatal care (ANC) utilization plays a vital role in improving both maternal and neonatal health outcomes (1). It provides pregnant women with the opportunity to engage with healthcare providers on key aspects such as proper nutrition, recognition of obstetric danger signs, and development of a birth preparedness plan (2). Additionally, ANC facilitates access to essential preventive interventions, including iron and folic acid supplementation and tetanus toxoid vaccination (3). Utilizing high-quality ANC services marked by timely initiation and regular follow-up visits has the potential to decrease pregnancy-related maternal mortality by approximately 20% (4). Most components of ANC services experience minimal expenses for the pregnant women (5, 6).

**Figure 1:**
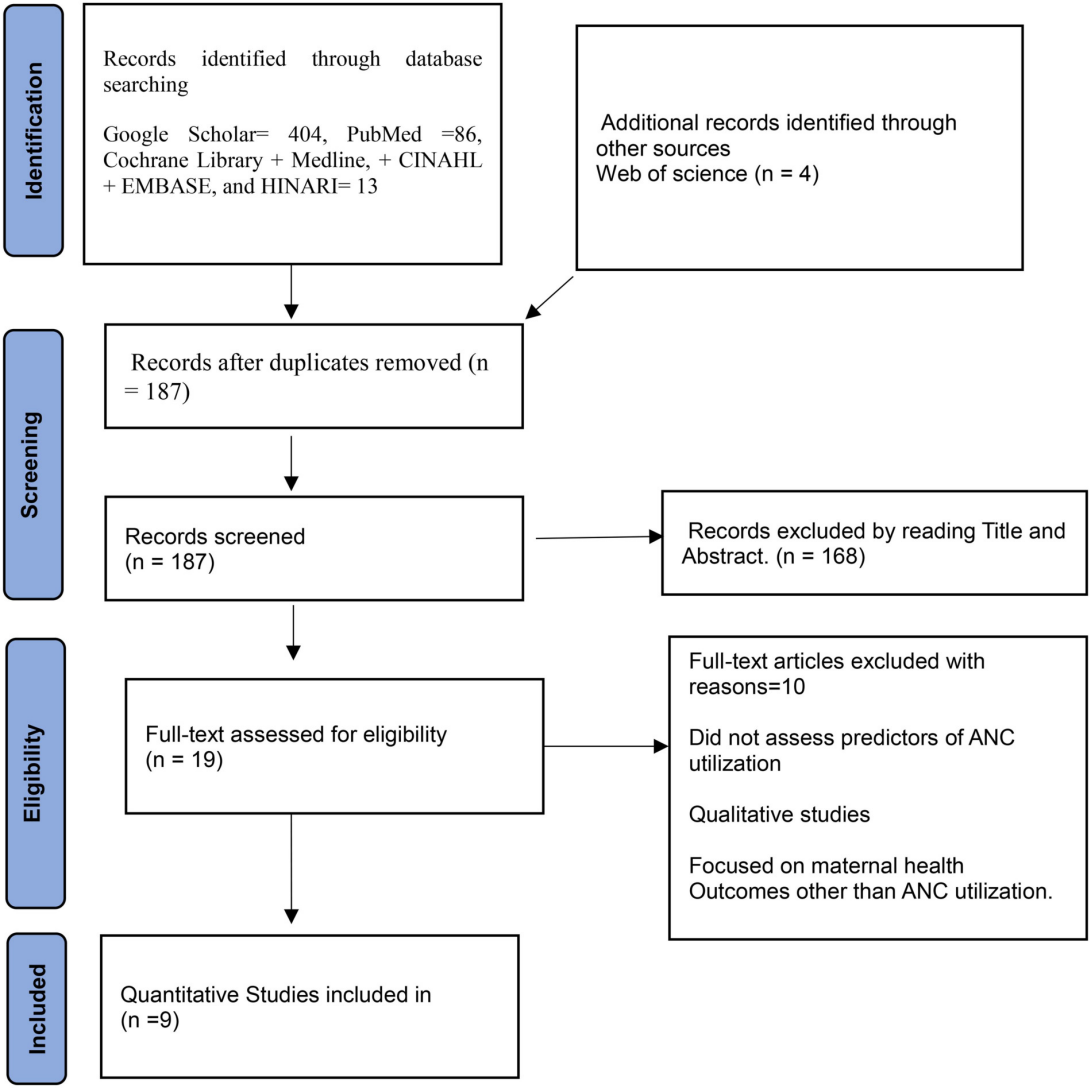
Flow diagram illustrating the selection and inclusion of studies in the review.

Worldwide, the utilization rate of ANC services is estimated at 72.9%, with coverage varying from 53.3% and 93.3% in developing and developed countries respectively (2). In regions with the highest maternal mortality rates, particularly in sub-Saharan Africa, only about 52% of women attend the recommended minimum of four antenatal care visits (7). This proportion is even lower in humanitarian settings with 25% in Somalia (7). ANC represents one of the most cost-effective approaches to enhancing maternal, newborn, and child health (MNCH) in low- and middle-income countries (LMICs). Despite substantial evidence linking ANC visits to significant health and economic advantages, millions of expectant mothers particularly in LMICs still fail to seek ANC services at health facilities. In fact, an estimated 10%–15% of pregnant women in these regions do not receive even a single ANC visit throughout pregnancy (8–10). According to the 2019 Mini Ethiopian Demographic and Health Survey (EDHS), ANC service utilization rose markedly, increasing from 28% in 2005 to 74% in 2019 (11).

Somalia has an average fertility rate of 6.9 children per woman (12). According to the 2020 Somali Health and Demographic Survey (SDHS), women in the country face a lifetime maternal death risk of one in 22, with a maternal mortality ratio estimated at 692 deaths per 100,000 live births—one of the highest globally (12). The SDHS reported that 31% of women aged 15–49 who had a live birth received ANC from skilled health professionals during their most recent pregnancy, while only 24% attended the recommended minimum of four ANC visits (13). Only 17% of women received two or more tetanus toxoid injections during their most recent pregnancy (14), whereas a striking 89% of mothers did not undergo any postnatal examination within the first two days following delivery (12). In terms of delivery location, approximately 79% of births occurred at home, predominantly assisted by traditional birth attendants, while only about 21% took place in health facilities.

Several predictors have been identified as contributors to the low utilization of antenatal care (ANC) services. These predictors can be systematically classified into socioeconomic and demographic factors, obstetric characteristics, health system and organizational constraints, service quality-related factors, infrastructural inadequacies, socio-cultural influences, and transportation barriers particularly prevalent in rural and hard-to-reach areas (15–19). A study in Somaliland identified maternal education, employment status, place of residence, wealth index, husband's education and employment, exposure to mass media, region, and pregnancy intention as significant predictors of antenatal care utilization (20). Another study conducted in southern and central Somalia identified marital status, occupation, household breadwinner, and distance to the health facility, encouragement to attend ANC, and availability of a midwife in the health facility as significant predictors of antenatal care utilization (7). Also a study conducted in Puntland, Somalia identified gravida status, ANC visit frequency, meal frequency, and household food insecurity as significant predictors of antenatal care utilization (21). ANC utilization within Somalia remains scarce, creating a significant gap in the literature that impedes effective efforts to strengthen health systems (22, 23). Few studies have addressed predictors of antenatal care utilization in Somalia (24). To the best of our knowledge, no prior systematic review has specifically synthesized evidence on this topic within the Somali. Thus, the aim of this review was to systematically examine the factors associated of antenatal care service utilization among women of reproductive Age in Somalia.

## Materials and methods

### Study selection

The systematic review followed the Preferred Reporting Items for Systematic Reviews and Meta-Analyses (PRISMA) guidelines (25) and was registered in the PROSPERO database (registration number: CRD420251244002). Articles retrieved from the listed electronic databases were first screened by title, and abstract to determine relevance. Studies that met the inclusion criteria were then subjected to a full-text review. Additionally, reference lists of the included articles were examined to identify any other potentially relevant studies.

### Inclusion criteria

Studies were eligible for inclusion if they were quantitative (using primary or secondary data) and reported factors associated with ANC utilization based on multivariable analysis, conducted in Somalia and published between 2010 and 2025. ANC utilization in this review refers to attendance of at least one antenatal care visit during pregnancy. Various study designs, including cross-sectional, cohort, case–control, and longitudinal studies, were eligible for inclusion if they assessed predictors of ANC utilization (Figure 1).

### Exclusion criteria

Studies were excluded if they did not provide sufficient statistical evidence to determine independent predictors of ANC utilization. Publications that lacked accessible full texts, had incomplete methodological reporting, or did not clearly define ANC utilization as an outcome variable were excluded. Research articles such as editorials, reviews, commentaries, and conference summaries were excluded because they do not provide extractable empirical data. Studies conducted outside Somalia were excluded to maintain contextual relevance. Additionally, studies focusing on broader maternal health outcomes where ANC utilization could not be disaggregated as a distinct analytical outcome were excluded to preserve conceptual clarity.

### Quality assessment

Quality assessment of the studies included in this review was conducted by the lead reviewer in collaboration with the co-authors. The Quality Assessment Tool for Observational Cohort and Cross-Sectional Studies was used to evaluate methodological quality, as applied in previous systematic review (26). This tool comprises 14 items that assess key methodological components, including study objectives, study population, sampling methods, sample size, and statistical analysis. Each item was rated as “Yes” (1) or “No” (0), with additional response options of cannot determine (CD), not applicable (NA), **or** not reported (NR). All studies included in the review were appraised using this standardized quality assessment approach. The included studies were conducted across multiple regions of Somalia, including Somaliland, Puntland, Banadir Region, Mudug Region, and Southern and Central Somalia. All studies were cross-sectional designs, including one hospital-based retrospective study and one secondary analysis of the Somaliland Demographic and Health Survey (SLDHS 2020). Sample sizes ranged from 60 to 3,375 participants, indicating considerable variability in study scope and population coverage. The strength and consistency of the reported predictors may be influenced by variations in study quality. Studies with lower methodological rigor are more likely to yield biased or inconsistent results (27). Accordingly, differences in study design, sample size, analytical approaches, and measurement reliability may partly explain the observed variability in reported predictors across the literature.

### Search strategy

All relevant studies were systematically searched from international databases using key terms such as predictors, determinants, antenatal care, maternal health care, prenatal care, utilization, associated factors, women of reproductive age, and Somalia. The databases searched included: Google Scholar, PubMed, Medline, CINAHL, EMBASE, and African Journal Online covering studies conducted in Somalia between 2010 and 2025. The PubMed search strategy was as follows: (((((((Antenatal care) OR (Maternal health care)) OR (Prenatal care)) AND (Utilization)) OR (Predictors)) AND (Determinants)) OR (Associated factors)) AND (Women of reproductive age)) AND (Somalia). Additionally, the reference lists of retrieved studies were reviewed to ensure that no relevant publications were missed.

### Data extraction

Data were extracted from all eligible studies using a structured extraction tool developed by the research team based on predefined inclusion and exclusion criteria. The tool was collaboratively designed by all authors to ensure it captured the necessary information to address the review objectives. Two reviewers (NA and SA) independently extracted data from each study. Extracted details included the author, year of publication, study location, design, sample size, and identified predictors of antenatal care utilization (see Table 1).

**Table 1:** Summary of articles included in the review.

Author, year	location	Design	Sample size
Abdikarim et al. (20)	Somaliland, Somalia	Cross-sectional	2,741
Jimale et al. (28)	Banadir Region, Somalia	Cross-sectional study	407
Miikkulainen, et al. (7)	Southern and Central Somalia	Cross-sectional study	792
Feiruza Mohammed et al. (21)	Puntland, Somalia	Cross-sectional study	361
Abdullahi Muse Mohamoud, et Al. (29)	Benadir Region, Somalia	Descriptive cross-sectional, retrospective hospital-based	60
Mouhoumed et al. (30)	Borama, Awdal Region, Somaliland	Cross-sectional study	330
Haji et al. (31)	South Gaalkacyo District, Mudug Region, Somalia	Cross-sectional study	450
Abdiwali et al. (32)	Somaliland, Somalia	Cross-sectional (SLDHS 2020 secondary data analysis)	3,192
Abdi et al*.* (33)	Somalia	Cross-sectional study	3,375

## Results

### Socio-demographic factors

#### Maternal age

Only one study identified maternal age as a significant predictor of ANC use. Women aged 25–35 years were nearly three times more likely to initiate ANC early and to complete at least four ANC visits compared to women older than 35 years (30) (Table 2).

**Table 2:** Predictors of antenatal care frequency, utilization and timing.

Factors	Predictors	<4 ANC visit	>4 ANC visits	Initiation of the first ANC visit
Sociodemographic factors	Maternal Age	(30)	(30)	(30)
	Maternal education		(20, 31, 32)	
	Maternal residency		(20, 32, 33)	
	Occupation	(28)	(20, 28, 31, 32)	
	Marital status	(28)	(7, 28, 32)	
	Family wealth index		(20, 32, 33)	
	Mass media exposure		(20)	
Maternal knowledge and psychosocial factors	Maternal intention		(20)	
	Knowledge of ANC		(29–31)	(30)
Health system and access–related factors	Distance to health facility		(7)	
	Accessibility to the health facility		(31)	
	Perceived health care workers’ attitude		(31)	
Obstetric and pregnancy-related factors	Gestational age		(29, 30)	(30)
	Gravida	(28)	(21, 28–30)	(30)

#### Maternal education

In three studies, an association between maternal education and ANC utilization was reported. One study found that women with primary and secondary education were 1.5 and 1.67 times more likely, respectively, to utilize ANC services compared to those with no formal education (20). The remaining two studies also reported a significant association between maternal education and ANC utilization (31, 32) (Table 2).

#### Maternal residency

In three studies, maternal residence was reported as a significant factor influencing ANC utilization. Two studies found that women residing in rural areas were less likely to utilize ANC services compared to those living in urban areas (20). Conversely, one study reported that women living in urban areas were more likely to access ANC services than their rural counterparts (32, 33) (Table 2).

#### Occupation

Four studies reported on the role of occupational status in ANC attendance. Across three studies, mothers who were employed were found to be less likely to attend at least four ANC visits (28, 31, 32). In contrast, one study reported that women whose husbands had worked in the last 12 months were more likely to utilize ANC services (20) (Table 2).

#### Marital Status

Three studies reported marital status as a predictor of ANC use. Divorced, widowed, or separated women were less likely to attend ANC or complete four visits compared to married women (7, 28, 32) (Table 2).

#### Family wealth index

Three studies reported a significant association between wealth status and ANC service utilization. Women from richer or richest households had higher odds of initiating ANC early and completing ≥4 ANC visits compared to those in lower wealth categories (20, 32) (Table 2).

## Reproductive and pregnancy-related factors

### Gravida status

Four studies reported a relationship between gravida and ANC use. Primigravida women were more likely to attend ANC early, whereas multigravida women showed varied patterns depending on parity and previous pregnancy experiences (21, 28–30) (Table 2).

### Gestational age at first visit

Two studies found that women who presented during the **first trimester** (<3 months) were more likely to achieve recommended ANC visits than those who initiated care later in pregnancy (29, 30) (Table 2).

### Maternal intention

One study found that pregnancy intention significantly influenced ANC utilization. Women who intended their pregnancies showed more likely to ANC utilization (20) (Table 2).

## Knowledge and behavioral factors

### Knowledge of ANC

Three studies reported that inadequate knowledge of ANC services was associated with poor ANC utilization. Women with better knowledge were more likely to initiate ANC early and meet the recommended number of visits (29–31) (Table 2).

### Mass Media exposure

One study revealed that women with exposure to mass media were more likely to utilize ANC services than those without media exposure (20) (Table 2).

## Health system and accessibility factors

### Health worker attitude

One study found that women who perceived health workers' attitudes as unfriendly were less likely to attend ANC visits (31) (Table 2).

### Accessibility to health facility

One study showed that women who reported having good access to a health facility were significantly more likely to utilize ANC services than those not reported (31) (Table 2).

### Distance to health facility

One study reported that greater distance to a health facility was associated with lower ANC utilization (7) (Table 2).

## Discussion

This systematic review examined the Predictors of Antenatal Care Service Utilization among Women of Reproductive Age in Somalia. The National Review Report published in 2022 identified several factors contributing to the high maternal mortality rate in Somalia, including low utilization of antenatal and a limited proportion of deliveries occurring in health facilities or attended by skilled healthcare providers (34). Furthermore, prolonged conflict, along with related challenges such as poverty and limited educational opportunities, has resulted in a persistent shortage of midwives in the country (7). The findings revealed that ANC use is associated with a complex interaction of sociodemographic, obstetric, knowledge-related, and health-system factors. Overall, the review highlights persistent inequities in ANC utilization and identifies several potentially modifiable factors associated with utilization.

This review, the maternal age was identified as a factor associated with ANC utilization. Women aged 25–35 years were more likely to initiate ANC early and complete the recommended number of visits compared to older women. Similar reviews conducted in Iran and Ethiopia have also reported that younger women are more likely to initiate ANC early (35, 36). In contrast, a review from sub-Saharan Africa found that increasing maternal age was associated with a higher likelihood of attending at least one ANC visit (37). However, a systematic review examining factors influencing ANC utilization in Ethiopia reported that maternal age was not significantly associated with ANC us (38).

Maternal education showed a strong and consistent association with ANC utilization. Women with primary or secondary education were significantly more likely to use ANC services compared to those with no formal education. Which aligns two systematic reviews from low- and middle-income countries found that higher levels of women's education were associated with increased use of maternal health services, including antenatal care (39, 40). This suggests that higher female education levels are associated with greater ANC utilization, although causal relationships cannot be established from cross-sectional evidence. In the systemic review residence was another associated factor. Women living in rural areas were consistently less likely to utilize ANC compared to urban residents. This finding aligns with evidence from studies conducted in Ghana and Nigeria, which similarly showed that women residing in urban areas were more likely to utilize antenatal care services compared to their counterparts in rural settings (41, 42).

In this review, occupational status presented mixed findings. In three studies, employed mothers were less likely to attend at least four ANC visits, possibly due to time constraints, inflexible work schedules, or socioeconomic pressures. Which is consistent another study that reported employment was found to hinder ANC utilization, with women often placing greater value on meeting work responsibilities for economic reasons than on attending ANC appointments (43).

Obstetric history also played a significant role in this review. Primigravida women were more likely to attend ANC early, while multigravida women showed varying patterns depending on previous pregnancy experiences. The reduced utilization observed among women with higher parity may be associated with factors such as childcare responsibilities, financial constraints, or prior experiences with ANC services. Additionally, women with previous pregnancies may perceive themselves as experienced, which may be linked to delayed initiation of ANC (44). Also women with previous pregnancies may view themselves as already experienced and familiar with routine ANC care, which can lead them to delay starting antenatal visits and reduce the number of contacts they make (45).

Early gestational age at first visit was consistently associated with completion of the recommended number of ANC visits in the current review. Women initiating care in the first trimester had a higher likelihood of achieving ≥4 visits. Early initiation has been consistently associated with completion of the recommended number of ANC visits, and delays often lead to missed opportunities for screening, prevention, and timely management. This aligns with earlier studies from Ethiopia showing that women who believed ANC should begin in the first trimester were more likely to start care early compared with those who thought it should begin later in pregnancy (46, 47).

Knowledge of ANC was associated with higher utilizationin several studies. Women with inadequate understanding of ANC services were less likely to initiate early or complete recommended visits. This may be due to awareness of pregnancy danger signs as well as knowledge about the appropriate timing and recommended number of ANC visits was increased of attending at least one ANC visit (16, 17).

Marital status and pregnancy intention also contributed to ANC utilization patterns in the current review. Married women and those with intended pregnancies were more likely to access ANC services.This association may reflect differences in social support or pregnancy planning, although causal pathways cannot be inferred. Likewise, the positive influence of intended pregnancy on the use of maternal healthcare services has been documented in several studies conducted in other settings (48–50). Another study also revealed that married women were more likely to utilize antenatal care services compared with those who had never been married or were not currently married (51).

According to the review, key factors associated with service utilization included

health worker attitudes, accessibility of health facilities, distance to health facilities, and the family wealth index. Similarly, a study conducted in Nigeria reported that several barriers such as lack of financial resources, poor-quality healthcare, transportation difficulties, unfriendly attitudes of healthcare providers, and spousal disapproval can prevent rural Nigerian women from accessing evidence-based maternal and health services (52).

## Conclusion

This systematic review demonstrates that antenatal care utilization in Somalia is influenced by a complex interplay of sociodemographic characteristics, obstetric history, knowledge and awareness, socioeconomic status, and health system accessibility. Maternal education, place of residence, wealth index, marital status, pregnancy intention, early initiation of ANC, and proximity to health facilities consistently emerged as key associated factors of ANC utilization, frequency, and timing. These findings highlight persistent inequities in access to maternal health services, particularly among rural, poorer, less educated, and multiparous women. The findings suggest that multi-sectoral and context specific approaches may help improve ANC utilization in Somalia. Strategies such as expanding female education, strengthening community health education, improving physical and financial access to services, and addressing health system constraints could potentially enhance early initiation and continuity of ANC. These recommendations should be considered as evidence informed hypotheses rather than confirmed solutions, as most included studies were cross sectional. Further longitudinal and intervention based research is needed to determine their effectiveness in improving maternal and neonatal health outcomes.

## Strengths and limitations

This review followed PRISMA guidelines and applied a comprehensive search strategy across multiple databases, enhancing the rigor and transparency of the review process. Also the inclusion of only quantitative studies with multivariable analyses strengthened the reliability of the findings by accounting for potential confounding factors and providing robust evidence on predictors of ANC utilization.

The majority of the included studies were cross-sectional in design, which limits the ability to infer causal relationships between the identified factors and antenatal care (ANC) utilization. As a result, the reported predictors should be interpreted as associations rather than evidence of causality. Additionally, heterogeneity in outcome definitions particularly regarding the number and timing of ANC visits may have affected comparability across studies and contributed to variability in reported findings. These methodological limitations should be considered when interpreting the strength, consistency, and policy implications of the identified predictors.

## Data Availability

The original contributions presented in the study are included in the article/Supplementary Material, further inquiries can be directed to the corresponding author.
